# Treatment of the Carotid In-stent Restenosis: A Systematic Review

**DOI:** 10.3389/fneur.2021.748304

**Published:** 2021-10-04

**Authors:** Hao Huang, Lingshan Wu, Yinping Guo, Yi Zhang, Jing Zhao, Zhiyuan Yu, Xiang Luo

**Affiliations:** Department of Neurology, Tongji Hospital, Tongji Medical College, Huazhong University of Science and Technology, Wuhan, China

**Keywords:** in-stent restenosis (ISR), treatment, percutaneous transluminal angioplasty (PTA), carotid endarterectomy (CEA), carotid artery stent (CAS)

## Abstract

**Background and Purpose:** In-stent restenosis (ISR) after carotid artery stent (CAS) is not uncommon. We aimed to evaluate therapeutic options for ISR after CAS.

**Methods:** We searched PubMed and EMBASE until November 2, 2020 for studies including the treatment for ISR after CAS.

**Results:** In total, 35 studies, covering 1,374 procedures in 1,359 patients, were included in this review. Most cases (66.3%) were treated with repeat CAS (rCAS), followed by percutaneous transluminal angioplasty (PTA) (17.5%), carotid endarterectomy (CEA) (14.3%), carotid artery bypass (1.5%), and external beam radiotherapy (0.4%). The rates of stroke & TIA within the postoperative period were similar in three groups (PTA 1.1%, rCAS 1.1%, CEA 1.5%). CEA (2.5%) was associated with a slightly higher rate of postoperative death than rCAS (0.7%, *P* = 0.046). Furthermore, the rate of long-term stroke & TIA in PTA was 5.7%, significantly higher than rCAS (1.8%, *P* = 0.036). PTA (27.8%) was also associated with a significantly higher recurrent restenosis rate than rCAS (8.2%, *P* = 0.002) and CEA (1.6%, *P* < 0.001). The long-term stroke & TIA and recurrent restenosis rates showed no significant difference between rCAS and CEA.

**Conclusions:** rCAS is the most common treatment for ISR, with low postoperative risk and low long-term risk. CEA is an important alternative for rCAS. PTA may be less recommended due to the relatively high long-term risks of stroke & TIA and recurrent restenosis.

## Introduction

Carotid artery stenosis accounts for 10 to 15% of ischemic stroke (1). Carotid endarterectomy (CEA) remains a reference treatment for carotid stenosis. Carotid artery stenting (CAS) became an important alternative to CEA (2). In CAS, the periprocedural risk and the in-stent restenosis (ISR) risk in the long term are major concerns (3, 4). Technical advances, including the development of embolic protection devices and stents, have helped to reduce the periprocedural risk. A meta-analysis of five randomized trials that exclusively used embolic protection devices showed that CAS and CEA were associated with a similar risk of a composite of periprocedural stroke, myocardial infarction, death, or non-periprocedural ipsilateral stroke (5). However, ISR after the CAS is still an issue. A recent Cochrane meta-analysis showed that CAS had a higher risk of moderate or higher restenosis (>50%) or occlusion than CEA and a similar risk of severe restenosis (>70%) with CEA (6). A systematic review showed that the >50% restenosis rate at 6 months was 3.9%, 12 months was 5.7% (7). The second analysis of the International Carotid Stenting Study (ICSS) showed that the cumulative 5-year risk of >50% restenosis was 40.7% and the cumulative 5-year risk of >70% restenosis was 10.6% (8). Therefore, the in-stent restenosis after CAS is an important problem to address. Some options, including percutaneous transluminal angiography (PTA) with balloon, repeat CAS (rCAS), CEA and artery bypass, have been proposed and compared in previous reviews (9–11). Recently, new techniques, such as drug-eluting balloons and stents, were proposed (12, 13). Here, we provided an updated systematic review of the current literature to evaluate the therapeutic options for ISR after CAS.

## Methods

### Search Strategy

A literature search in the PubMed and EMBASE databases was conducted to locate relevant publications until November 2, 2020. We referred the systematic reviews and meta-analyses of this field in recent years (10, 14, 15). The search terms and phrases used in this article were “carotid arteries”, “carotid artery stent”, “in-stent restenosis” and “therapeutics”. The full search strategy is listed in Table 1.

**Table 1:** Search terms in PubMed and EMBASE.

**Database**	**Searches**
PubMed	Search 1: “Carotid Arteries” (MeSH) OR (Arteries, Carotid) OR (Artery, Carotid) OR (Carotid Artery)
	Search 2: (carotid artery stent*) OR (CAS) OR (carotid stent*) OR (carotid artery stent angioplasty) OR (carotid artery stent placement) OR (carotid stent placement)
	Search 3: (in-stent restenosis) OR (in-stent re-stenosis) OR (in-stent stenosis) OR (stent restenosis) OR (stent stenosis) OR (ISR)
	Search 4: “Therapeutics” [Mesh] OR (Therapeutic) OR (Therap*) OR (Treatment*)
	(Search 1) AND (Search 2) AND (Search 3) AND (Search 4)
EMBASE	Search 1: (exp Carotid Artery) OR “Carotid Arteries” OR “carotid blood vessel” OR “carotid”
	Search 2: (exp carotid artery stenting) OR “carotid artery stent*” OR “CASS” OR “carotid stent*” OR “carotid angioplasty and stent*” OR “carotid artery stent angioplasty” OR “carotid artery stent placement” OR “carotid stent placement”
	Search 3: (exp in-stent restenosis) OR “in-stent re-stenosis” OR “in-stent stenosis” OR “stent restenosis” OR “stent stenosis” OR “ISR” OR “stent obstruction” OR “stent occlusion”
	Search 4: (exp Therapy) OR “Therapeutic*” OR “Therap*” OR “Treatment*”
	(Search 1) AND (Search 2) AND (Search 3) AND (Search 4)

### Study Selection

**Figure 1:**
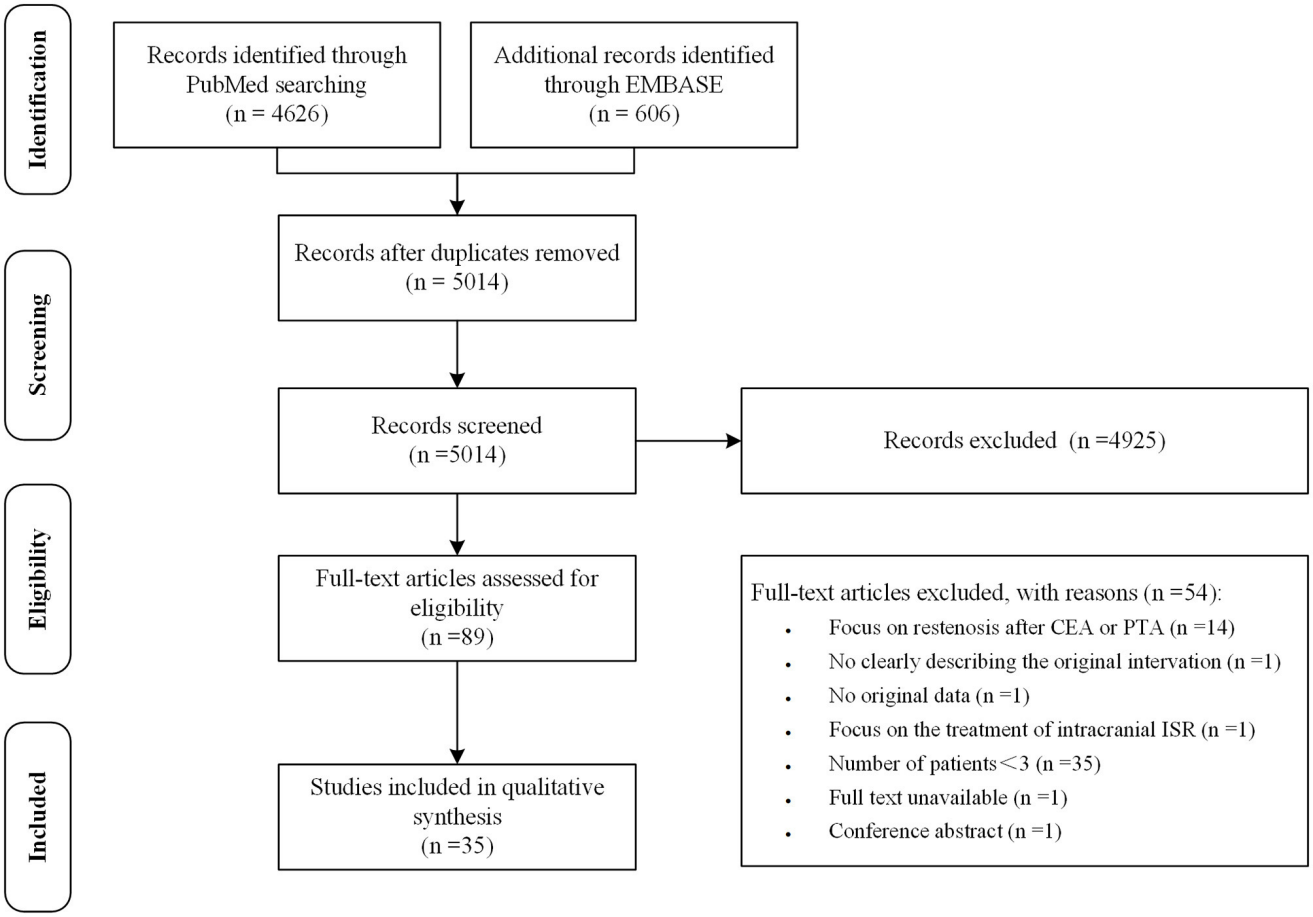
The preferred reporting items for systematic reviews and meta-analyses flowchart of the study selection.

After removal of duplicates, the initial literature search identified 5014 records. Studies were first screened based on the title and abstract. Studies in which a specific treatment for ISR and its outcome were described were included. Studies that were published in a language other than English or Chinese were excluded. After reviewing titles and abstracts, 89 articles were retained for full-text review. Articles with two or fewer subjects, without original data, focusing on restenosis after CEA or PTA, or without available full texts were excluded. This narrowed the data base to 35 studies. The process of study selection was independently performed by two authors (Lingshan Wu and Hao Huang) according to the Preferred Reporting Items for Systematic Reviews and Meta-Analyses guidelines (Figure 1) (16). Disagreements were resolved by discussion and where necessary, a third reviewer with expertise in the field (Xiang Luo) was consulted. Data of demographics, selection criteria of study population, imaging techniques, procedure for ISR, protocol of follow-up and outcomes were extracted.

### Critical Quality Evaluation

For critical quality evaluation, we extracted characteristics and selection criteria of patients, imaging techniques and procedure for ISR, follow-up protocol, and outcomes of treatment.

### Statistical Analysis

We reported our findings using descriptive statistics. Continuous data are presented as the mean ± SD. When calculating the proportion of patients with a certain characteristic or an outcome event, the denominator was the number of patients who were reported in the included studies. The evaluation of the post-operative and long-term outcome was based on the number of patients with reported data. Data were analyzed using SPSS 25. To compare characteristics and rates of outcomes between patients treated with PTA, rCAS, and CEA, Chi-square test and Fisher exact test were used. A value of *p* < 0.05 (two-tailed) was considered statistically significant.

## Results

### Quality Assessment of the Selected Articles

Details on quality criteria of the 35 selected articles are provided in Supplementary Table I (online only). Among the 35 selected studies, 19 studies did not describe the characteristics of patients, 3 did not describe the selection criteria of study population, 4 did not describe the imaging techniques used for the diagnosis of ISR, 14 did not clearly describe the procedure for ISR, 11 did not report the postoperative outcome, 17 did not clearly describe the follow-up protocol and 2 did not report the follow-up outcomes.

### Baseline Characteristics

The included articles described 1,374 procedures in 1,359 patients. The average age of participants was 68.68 ± 4.79 years. Gender was not reported in 33% of subjects. Where gender was reported 33% were male. Overall, 523 (38%) patients were symptomatic, 333 (25%) patients were asymptomatic, and 11 studies did not report patient symptoms. Risk factors were not reported in 19 studies. In the remaining 16 studies, 737 (84%) patients had hypertension, 340 (39%) had diabetes mellitus, 113 (50%) had hyperlipidemia, 276 (35%) had coronary artery disease, 291 (38%) smoked, and 108 (15%) had significant carotid artery ISR with contralateral occlusion. Of the 35 studies included in this study, 5 studies (17–21) reported 12 patients with the history of radiation therapy. The clinical characteristics of the patients are detailed in Supplementary Table II (online only).

### Imaging Technique

**Figure 2:**
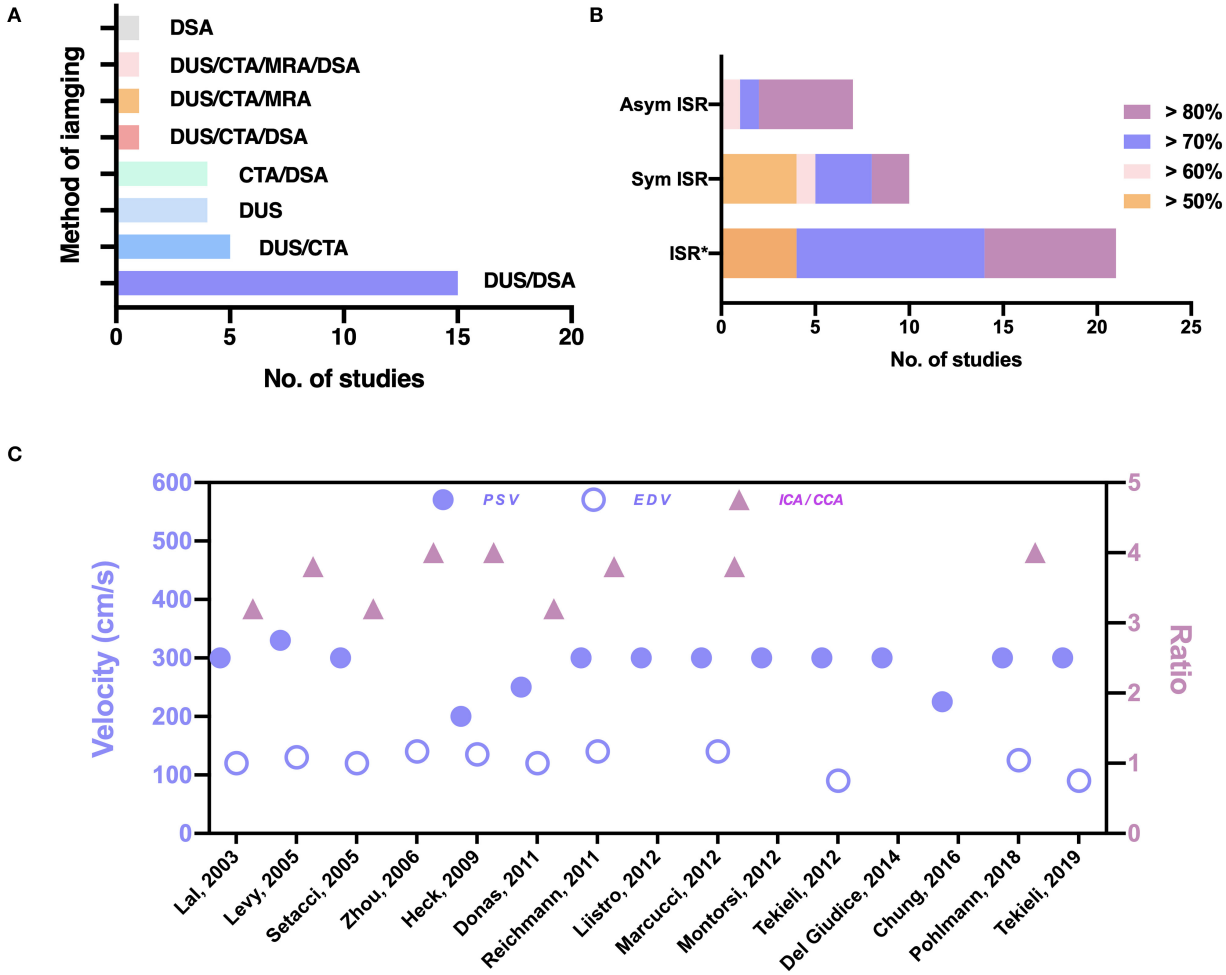
The imaging techniques and indications in the 35 studies. **(A)** Imaging techniques used for the diagnosis of ISR. **(B)** Criteria for the treatment of ISR. Asterisks (^*^) indicate a category of ISR not reported in the studies. **(C)** Thresholds of PSV, EDV, and ICA/CCA the in diagnosis of ISR. Sym, symptomatic; Asym, asymptomatic; DUS, duplex ultrasonography; DSA, digital subtraction angiography; CTA, CT angiography; MRA, MR angiography; EDV, end diastolic velocity; PSV, peak systolic velocity; ICA/CCA, internal to common carotid artery peak systolic velocity ratio.

The ISR criteria, the imaging technique, and the duplex ultrasonography (DUS) thresholds are shown in Figure 2 (details are listed in Supplementary Table III online only). DUS is useful for follow-up after carotid intervention for the detection of restenosis. Four (11%) studies did not report the type of imaging tools employed in making the diagnosis of ISR. In most studies, DUS was used as a primary screening tool for ISR (Figure 2A). To confirm the diagnosis, digital subtraction angiography (DSA) was performed in 15 (43%) studies. In five (14%) studies, computed tomographic angiography (CTA) was performed; in four (11%) studies CTA or DSA was performed; in one (3%) study CTA or magnetic resonance angiography (MRA) was performed; and in one (3%) study CTA or MRA or DSA was performed to provide additional confirmation of the diagnosis. In four (11%) studies only DUS was used; in one (3%) study only DSA was used; and in one (3%) study CTA or DSA were used as diagnostic tools (Figure 2A). The DUS criteria for ISR were reported in 15 studies, and ISR was defined as >70% or 80% in most studies; in one study it was defined as > 50% (17). For high grade restenosis (ISR > 70% or 80%), the peak systolic velocity (PSV) threshold was relatively consistent at 300–330 cm/s, except for three studies where the PSV threshold was 200, 225, and 250 cm/s (Figure 2C) (17, 22, 23). The end diastolic velocity (EDV) threshold was also consistent at 120–140 cm/s, except for two studies where the EDV threshold was 90 cm/s (13, 24). The internal to common carotid artery peak systolic velocity ratio (ICA/CCA ratio) threshold varied from 3.2 to 4 (Figure 2C). The ISR criteria varied in the 35 studies (Figure 2B). Among asymptomatic patients, the inclusion criteria were ISR ≥ 60% in one study, ISR ≥ 70% in one study, and ISR ≥ 80% in five studies. Among symptomatic subjects, the inclusion criteria were ISR ≥ 50% in four studies, ISR ≥ 60% in one study, ISR ≥ 70% in three studies, and ISR ≥ 80% in two studies. Among studies that did not report the ISR category, the inclusion criteria were ISR ≥ 50% in four studies, ISR ≥ 70% in 10 studies, and ISR ≥ 80% in seven studies. Three studies did not report the inclusion criteria.

### Studies Characteristics

The characteristics of the 35 included studies were shown in Table 2. The number of patients included in these studies varied from 3 to 645. The time to ISR ranged between 5.3 months and 43.5 months, and the median time to ISR was 15.7 months.

**Table 2:** Summary of included studies.

**Study**	**Pts, No**.	**Sym ISR, No**.	**Time to ISR**	**ISR treatment, No**.	**Postoperative outcomes**		**Follow-up time**	**Long-term overall outcomes**		**Re-ISR, No**.
					**Stroke & TIA**	**Death**		**Stroke & TIA**	**Death**	
Chakhtoura et al., (18)	4	0	13 m	3 PTA, 1 rCAS	NR	0	10 m	0	0	0
Ehringer et al., (25)	8	1	6.7 m	6 rCAS, 3 PTA	0	0	NR	2	1	3
de Borst et al., (26)	4	2	8 m	4 CEA	0	0	13 m	0	0	0
Lal et al., (27)	5	NR	< 15 m	4 PTA, 1 rCAS	NR	0	NR	NR	0	2
Levy et al., (28)	6	3	16.4 m	4 PTA, 1 CB-PTA, 1 rCAS	0	0	23 m	1	0	2
Pokrajac et al., (29)	5	NR	NR	5 EBR	0	0	31.75 m	1	1	2
Raithel et al., (30)	22	NR	NR	20 PTA, 2 rCAS	NR	NR	NR	NR	NR	8
Setacci et al., (31)	14	5	39 m	3 PTA, 4 CB-PTA, 8 rCAS	0	0	12.4 m	0	0	0
Reimers et al., (32)	31	7	5.3 m	12 PTA, 10 CB-PTA, 10 rCAS	0	0	17m	0	0	1
Zhou et al., (19)	7	1	14 m	4 CB-PTA, 1 PTA, 2 rCAS	0	0	9 m	NR	1	2
Younis et al., (20)	11	3	18.3 m	2 PTA, 1 CB-PTA, 8 rCAS	0	0	10.3 m	0	0	1
Jimenez et al., (33)	4	1	43.5 m	2 CEA, 2 PTFE graft bypass	0	0	27.5 m	0	0	0
Juskat et al., (34)	3	2	11.2 m	1 PTA, 2 rCAS	NR	0	NR	0	0	0
Heck, (22)	6	NR	NR	6 CB-PTA	0	0	20 m	0	0	1
Zahn et al., (35)	86	NR	NR	53 rCAS, 33 PTA	NR	0	1 m	NR	NR	NR
Donas et al., (23)	16	6	7.8 m	12 PTA, 1 rCAS, 3 vein graft bypass	NR	NR	NR	NR	NR	5
Gonzalez et al., (36)	3	3	2 years	3 CEA	NR	0	1 y	NR	0	0
Reichmann et al., (21)	15	10	18.3 m	15 CEA	1	0	21 m	1	0	0
Jost et al., (37)	3	NR	20.5 w	3 CEA, 1 Dacron graft bypass	0	0	11.5 m	0	0	0
Liistro et al., (38)	3	0	NR	3 DEB-PTA	NR	0	23.3 m	0	0	0
Marcucci et al., (39)	7	3	13.1 m	5 CEA, 2 PTFE graft bypass	0	0	18 m	0	1	0
Montorsi et al., (40)	10	0	20.9 m	2 CB-PTA, 7 DEB-PTA, 1 rCAS	NR	NR	13.7 m	1	0	7
Tekieli et al., (24)	16	NR	38 m	16 PTA	NR	NR	8.7 m	1	NR	7
Del Giudice et al., (41)	9	9	3.6 m	9 DEB-PTA	0	0	36.6 m	0	1	3
Hynes et al., (42)	262	NR	NR	262 rCAS	2	1	NR	NR	NR	NR
Wu et al., (43)	21	21	15.7 m	15 CEA, 6 PTFE graft bypass	0	0	13.2 m	0	1	0
Chung et al., (17)	30	NR	NR	19 PTA, 21 rCAS	1	1	948 d	2	9	2
Columbo et al., (44)	8	4	NR	5 CEA, 3 bypass	0	0	38.7 m	2	3	1
Moon et al., (45)	14	NR	NR	4 PTA, 9 rCAS, 1 CEA	0	0	NR	6	NR	6
Nishihori et al., (46)	6	2	12 m	6 rCAS	0	0	19.2 m	NR	0	0
Arhuidese et al., (11)	645	426	NR	511 rCAS, 134 CEA	9	10	1 y	11	66	NR
Davidovic et al., (47)	4	4	26 m	4 bypass: 2 PTFE graft, 2 Dacron graft	NR	NR	13 m	0	0	0
Yu et al., (48)	10	9	6.5 m	10 CEA	0	0	25 m	0	1	1
Pohlmann et al., (12)	9	1	9 m	10 DEB-PTA	0	0	5 y	1	2	1
Tekieli et al., (13)	52	NR	22 m	19 PTA, 27 DEB-PTA,6 DES	1	0	NR	NR	NR	13

*Sym, symptomatic; Pts, patients; ISR, in-stent restenosis; Re-ISR, recurrent in-stent restenosis; NR, not report; PTA, percutaneous transluminal angioplasty; rCAS, repeat carotid artery stent; CEA, carotid endarterectomy; CB-PTA, cutting balloon PTA; DEB-PTA, drug-eluting balloon PTA; EBR, external beam radiotherapy; PTFE, polytetrafluoroethylene interposition graft; DES, drug-eluting stent; m, month(s); y, year(s)*.

### Treatment for ISR

Most cases were treated with rCAS (911, 66.3%), followed by PTA (240, 17.5%), CEA (197, 14.3%), carotid artery bypass (21, 1.5%), and external beam radiotherapy (EBR) (5, 0.4%). Of the 240 procedures, PTA was performed with a regular balloon in 156 (65%) cases, cutting balloon PTA (CB-PTA) was performed in 28 (11.7%) cases, and drug-eluting balloon PTA (DEB-PTA) was performed in 56 (23.3%) cases. Of the 911 rCAS cases, drug-eluting stenting (DES) was performed in 6 (0.4%) cases. Of the 21 carotid artery bypasses, 4 (19.0%) were performed with a reversed saphenous vein interposition graft, 13 (61.9%) with a polytetrafluoroethylene (PTFE) interposition graft, 3 (14.3%) with a Dacron interposition graft, and 1 (4.8%) with a woven heparin-bonded polyester graft.

### Postoperative Outcomes

As shown in Table 2 and Supplementary Table IV (online only), there were fourteen strokes & TIAs, twelve deaths, and thirty-two other events in the postoperative period. One stroke and two other events cannot be attributed to a definite treatment because they were not reported in detail. For the individuals treated with PTA, there was one stroke & TIA (1/93, 1.1%) and one death (1/183, 0.5%), all of which occurred following CB-PTA (1/26, accounting for 3.8% in both cases). For the individuals treated with rCAS, nine strokes & TIAs (9/844, 1.1%), six deaths (6/907, 0.7%), and eleven other events (five arrhythmias and six MIs, 11/850, 1.3%) occurred during the perioperative period. No death or other events occurred after DES. For the subjects treated with CEA, three strokes & TIAs (3/195, 1.5%), five deaths (5/198, 2.5%), and nineteen other events (one transient worsening of pre-existing paresis, three neck hematomas, one arrhythmia, four cranial nerve injuries, one dissecting aneurysm, one cerebral hyperperfusion, one tachyarrhythmia absoluta/atrial fibrillation with cardiac decompensation, one hypertensive urgency and tachyarrhythmia absoluta/atrial fibrillation, one hypoglossal nerve dysfunction and four myocardial infarctions, 19/189, 10.1%) occurred during the perioperative period. For the individuals treated with carotid artery bypasses, one case of pneumonia and one case of temporary dysfunction of the laryngeal nerve (2/8, 25%) occurred within the postoperative period, whereas no stroke & TIA or death occurred. In the EBR group, no complications occurred during the postoperative period.

### Long-Term Overall Outcomes

The duration of follow-up ranged from 30 days to 10 years. The long-term overall outcomes include postoperative events and events that occurred during follow-up. As shown in Table 2, there were 29 strokes & TIAs and 87 deaths. There were seven strokes and 11 deaths that cannot be attributed to a definite treatment because they were not reported in detail. For the patients treated with PTA, there were six strokes & TIAs (6/105, 5.7%) and five deaths (5/88, 5.7%). Among these events, one stroke & TIA (1/24, 4.2%) and one death (1/28, 3.6%) occurred after CB-PTA; two strokes & TIAs (2/29, 6.9%) and three deaths (3/29, 10.3%) occurred following DEB-PTA. Among the individuals treated with rCAS, there were ten strokes & TIAs (10/568, 1.8%) and 56 deaths (56/557, 10.1%). Among the subjects treated with CEA, there were four strokes & TIAs (4/188, 2.1%) and 12 deaths (12/176, 6.8%). One stroke & TIA (1/5, 20%) and one death (1/5, 20%) occurred after EBR. In the individuals treated with carotid artery bypass, there was one stroke & TIA (1/15, 6.7%) and two deaths (2/9, 22.2%).

### Recurrent In-stent Restenosis

There were 66 Re-ISR events noted in the studies; 37 events (37/133, 27.8%) occurred after PTA, five events (5/61, 8.2%) followed rCAS, one event (1/62, 1.6%) occurred after CEA, two events (2/5, 40%) occurred after EBR, and one event (1/18, 5.6%) occurred after carotid artery bypass. Among these events, four Re-ISR events (4/15, 26.7%) occurred after CB-PTA, 17 Re-ISR events (17/56, 30.4%) occurred following DEB-PTA, and one Re-ISR event (1/6, 16.7%) occurred following DES. Twenty Re-ISR events cannot be attributed to a definite treatment because they were not reported in detail. For the treatment of Re-ISR, rCAS was chosen in 22 (39.3%) cases, PTA in 21 (37.5%) cases, CEA with stent removal in six (10.7%) cases, and carotid artery bypass in six (10.7%) cases. Treatment for one (1.8%) case was unknown.

### Comparison of PTA, rCAS and CEA

For the postoperative risk, the rates of stroke & TIA (PTA 1.1%, rCAS 1.1%, CEA 1.5%) were similar in the three groups (*P* > 0.05 for all comparisons). In terms of deaths, subjects in the CEA group had a higher ratio compared with the rCAS group (2.5 vs. 0.7%, *P* = 0.046), whereas there was no difference between the PAT and CEA groups (0.5 vs. 2.5%, *P* = 0.217), nor between the PTA and rCAS groups (0.5 vs. 0.7%, *P* = 1). For the long-term overall outcomes, the proportion of stroke & TIA in the PTA group was higher than that in the rCAS group (5.7 vs. 1.8%, *P* = 0.036), while there was no significant difference between the PTA and CEA groups (5.7 vs. 2.1%, *P* = 0.198), nor between the rCAS and CEA groups (1.8 vs. 2.1%, *P* = 0.991). The rates of death (PTA 5.7%, rCAS 10.1%, CEA 6.8%) were similar in the three groups (P > 0.05 for all comparisons). Furthermore, patients in the PTA group had a higher ratio of Re-ISR events compared with the rCAS and CEA groups (27.8 vs. 8.2% and 1.6%, *P* = 0.002 and *P* < 0.001), while there was no difference between the rCAS and CEA groups (8.2 vs. 1.6%, *P* = 0.114). The comparison of outcomes among the three groups is shown in Table 3.

**Table 3:** Comparison of outcomes in the PTA, rCAS, and CEA groups.

**Outcome**	**PTA**	**rCAS**	**CEA**	**PTA vs. rCAS**		**PTA vs. CEA**		**rCAS vs. CEA**	
				**χ2**	***P***	**χ2**	***P***	**χ2**	***P***
**Postoperative outcomes**									
Stroke & TIA	1/93 (1.1%)	9/844 (1.1%)	3/195 (1.5%)	-	1	-	1	0.03	0.854
Death	1/183 (0.5%)	6/907 (0.7%)	5/198 (2.5%)	0	1	-	0.217	3.99	**0.046**
**Long-term overall outcomes**									
Stroke & TIA	6/105 (5.7%)	10/568 (1.8%)	4/188 (2.1%)	4.39	**0.036**	1.65	0.198	0	0.991
Death	5/88 (5.7%)	56/557 (10.1%)	12/176 (6.8%)	1.21	0.272	0.13	0.723	1.67	0.197
Re-ISR	37/133 (27.8%)	5/61 (8.2%)	1/62 (1.6%)	9.49	**0.002**	18.51	**<0.001**	-	0.114

*PTA, percutaneous transluminal angioplasty; rCAS, repeat carotid artery stent; CEA, carotid endarterectomy; Re-ISR, recurrent in-stent restenosis. Bold indicates statistical significance*.

## Discussion

Herein, a systematic review of 35 selected studies, including 1,359 patients and 1,374 treatments, of ISR after CAS demonstrated that the most used treatment was rCAS, followed by PTA and CEA. Techniques including carotid artery bypass and EBR were applied in few cases. New PTA techniques, including CB-PTA and DEB-PTA, were widely employed. DUS was the most common method in detecting ISR, and was usually accompanied with CTA, MRA, or DSA. PTA was associated with the highest long-term overall stroke & TIA and recurrent restenosis rates. The rates of postoperative and long-term stroke & TIA and recurrent restenosis showed no significant difference between the rCAS and CEA groups.

DUS is a non-invasive technique, which was used in many randomized controlled trials (RCTs), including CREST and EVA-3s (49–51). Previous data indicated that a PSV of 300–350 cm/s may be a good predictor of ≥ 70% ISR (52). The present study confirmed the applicability of a PSV threshold of 300–330 cm/s. Of note, DUS is an indirect method to detect the stenosis and results are operator- and stent-dependent (53). Previous studies also showed that restenosis detecting by DUS were usually overestimated in the stented artery (54, 55). Therefore, direct methods of detecting lumen stenosis, including DSA, CTA, and MRA, are important in confirmation of the diagnosis, especially DSA usually regarded as the gold standard. In addition, new image techniques, such as dual-source CTA (can address the effects of beam hardening in regular CTA) (56) and 3D black-blood MRI (can assess artery plaques) (57, 58), have been gradually applied to evaluate the artery stenosis. The present study found that criteria for restenosis treatment varied. The criteria for asymptomatic patients were stricter than that for symptomatic patients. This is consistent with intervention decisions for carotid artery stenosis (2). Although stenosis remains a primary consideration, treatment decisions may benefit from consideration of other parameters. One recent study showed that for asymptomatic carotid stenosis, high-risk plaques were common and associated with higher risk of ipsilateral ischemic cerebrovascular event, suggesting the degree of stenosis alone may not fully capture the clinical situation (59). For ISR, evaluation of the plaque, the stent and the vessel are important. Some new techniques, such as the intravascular optical coherence tomography (OCT) (60), make it possible to access the plaque, stent and vessel *in vivo* (61). OCT had been used to assess the carotid plaque characteristics in carotid disease (62). A study of coronary disease showed that OCT could help to identify high risk patients and guide the treatment of ISR (63). OCT as also applied to evaluate tissue prolapse after CAS (64). Given this, the use of OCT to evaluate restenosis after CAS should be considered in future. It should also be noted that almost one out of ten studies in the review did not report diagnostic modality to verify severity of the stenosis. All these limitations, especially the drawbacks of DUS, should be kept in mind when one intends to translate the current knowledge into clinical practice.

PTA was the most common method in a previous review (10), but in the present study, rCAS was the most frequently used treatment. With respect to the postoperative risk, PTA had the lowest risk. It should be noted that some studies, especially the study involving PTA, did not report the postoperative outcomes in detail, which could confound the results of our analysis. In the review, compared to rCAS, CEA was associated with higher rates of death and other complications, including bleeding and cranial nerve injury. A previous meta-analysis comparing the rCAS and CEA for the ISR after CAS found no difference in the mortality rate, stroke-free rate, and even the rate of cranial nerve injury and haematoma (14). Given the discrepancies in these findings, future studies should compare the postoperative risks after rCAS and CEA. With respect to the long-term overall risk, PTA was associated with the highest rates of stroke & TIA and recurrent restenosis. Our review also confirmed the high restenosis rate associated with PTA (10, 15). The death rate was relatively high in all subgroups. This may be attributed to the long duration of the follow up which ranged from several months and many years. The death causes included trauma, cancer, and the procedure. However, many studies didn't provide detailed clinical information of the cause and time of death. Thus, we should be cautious when interpreting the results.

CEA remains the gold standard for the treatment of carotid artery stenosis. CEA is also suitable for ISR, especially for the patients difficult to conduct rCAS and PTA (36, 65). A recent study comparing CAS and CEA in De Novo carotid stenosis and postintervention restenosis suggested that for post-CAS restenosis, CAS and angioplasty were recommended for moderate stenosis and CEA for severe stenosis (66). In the present analysis, CEA was associated with a relatively high rate of postoperative death but similar long-term overall risks of stroke & TIA, death, and recurrent restenosis as rCAS. These findings suggest that CEA is an acceptable alternative to rCAS, especially in difficult situations. The proportion of symptomatic ISR after PTA was significantly lower than that after rCAS and CEA (PTA 32%, rCAS 65%, CEA 66%), and the contralateral occlusion rate associated with PTA was higher than those associated with rCAS and CEA (PTA 50%, rCAS 14%, CEA 11%) (Supplementary Table VIII). This may reflect the preferred treatment choice in the real world.

New PTA techniques are seeing application including DEB-PTA, CB-PTA, and paclitaxel-coated balloon PTA. All these techniques are aimed at decrease the relatively high recurrent restenosis in PTA. A meta-analysis of RCTs comparing plain balloon angioplasty, DEB-PTA, and DES for the treatment of ISR in coronary intervention showed that DEB-PTA and DES were associated with a significantly lower restenosis risk than plain balloon angioplasty (67). In contrary to good effect of DEB-PTA in coronary artery disease, however, the present study demonstrated that the Re-ISR rates of these techniques were also high. As shown in Supplementary Table V (online only), the stent types employed in the studies included the Wallstent, Acculink, and Palmaz stents, and the drug-eluting stents were very few. These new treatment techniques should be further evaluated in the future.

## Limitation

There are some important limitations of the present study. First, some studies included in the review did not report basic information on patient symptoms or gender, indications, postoperative complications, or long-term outcomes in detail, which may cause publication bias. We also separately analyzed studies with intact basic information and found that the tendency of the comparisons between the outcomes of those interventions is similar to our original analysis but without statistical significance (Supplementary Table VI and VII). Second, there was heterogeneity in the imaging standards of ISR, the follow-up time, and clinical outcome identification among these studies. The sample size varied from 3 to 645 and the primary results were inevitably influenced by the studies with large sample size. Generally, pronounced heterogeneity within and between studies, a meta-analysis could not be conducted. Third, no study in the review was a RCT. Because of the heterogeneity and the data quality of these studies, we must be cautious when interpreting the results regarding the statistical evaluation of the postoperative and long-term outcome. We therefore cannot conclude which treatment for ISR is optimal based on the current evidence, and randomized controlled studies are needed to further assess these treatments.

## Conclusion

The indication for ISR depended on the degree of stenosis but the criteria for symptomatic and asymptomatic patients were different. DUS was the most common method used for detecting ISR and was often accompanied with CTA, MRA, or DSA. A PSV threshold of 300–330 cm/s correlated with high-grade ISR. Historically, rCAS is the most common treatment for ISR with low postoperative risk and low long-term risk. CEA is an important alternative for rCAS. PTA is less recommended due to its high long-term risks of stroke & TIA and recurrent restenosis. Based on the present analysis, it was not possible to ascertain which treatment for ISR is optimal and large, multi-center, blinded analysis may help stratify the optimum therapeutic options.

## Data Availability Statement

The original contributions presented in the study are included in the article/Supplementary Material, further inquiries can be directed to the corresponding author/s.

## Author Contributions

HH put forward the idea of this article, worked with LW to complete literature retrieval and screening, data extraction, and manuscript writing. HH and LW contributed equally to this work. XL participated in the guidance of experimental design, etc. YG, YZ, and JZ participated in the verification of the data included in these literatures. ZY participated in the language modification work of the article.

## Funding

Key Research and Development Program of Hubei Province (2020BCA070 to XL), the Application Foundation Frontier Special Project of Wuhan Science and Technology Bureau (2020020601012226 to XL), the Flagship Program of Tongji Hospital (2019CR106 to XL), and the second batch of clinician research projects of HUST to XL.

## Conflict of Interest

The authors declare that the research was conducted in the absence of any commercial or financial relationships that could be construed as a potential conflict of interest.

## Publisher's Note

All claims expressed in this article are solely those of the authors and do not necessarily represent those of their affiliated organizations, or those of the publisher, the editors and the reviewers. Any product that may be evaluated in this article, or claim that may be made by its manufacturer, is not guaranteed or endorsed by the publisher.
