# Supplementary material for: Treatment of the Carotid In-stent Restenosis: A Systematic Review
Source: Front Neurol. 2021 Oct 4;12:748304. doi: 10.3389/fneur.2021.748304 (PMC8521022; doi:10.3389/fneur.2021.748304)
Supplement: Supplementary file 1 [file Table_1.DOCX]

**Supplementary Table I (online only).** Quality assessment of the 35 selected articles.

| Study | Characteristics of patients described | Selection criteria of study population clearly described | Imaging techniques used for the diagnosis of ISR clearly described | Proceduce for ISR clearly described | Postoperative outcome evaluated | Follow-up protocal described | Follow-up outcomes evaluated |
| --- | --- | --- | --- | --- | --- | --- | --- |
| Chakhtoura et al, (21), 2001 | √ | √ | √ | × | × | √ | √ |
| Ehringer et al, (22), 2002 | √ | √ | √ | √ | √ | × | √ |
| de Borst et al, (23), 2003 | × | √ | √ | × | √ | × | √ |
| Lal et al, (24), 2003 | × | √ | √ | × | × | × | √ |
| Levy et al, (25), 2005 | × | √ | √ | × | √ | × | √ |
| Pokrajac et al, (26), 2005 | × | √ | √ | √ | √ | × | √ |
| Raithel et al, (27), 2005 | × | √ | × | × | × | × | √ |
| Setacci et al, (28), 2005 | √ | √ | √ | × | √ | √ | √ |
| Reimers et al, (29), 2006 | √ | √ | √ | √ | √ | × | √ |
| Zhou et al, (30), 2006 | √ | √ | √ | × | √ | × | √ |
| Younis et al, (31), 2007 | × | √ | √ | × | √ | × | √ |
| Jimenez et al, (32), 2008 | × | √ | √ | √ | √ | √ | √ |
| Juskat et al, (33), 2008 | × | × | √ | × | × | × | √ |
| Heck, (18), 2009 | × | √ | √ | √ | √ | √ | √ |
| Zahn et al, (34), 2010 | × | √ | × | × | × | √ | × |
| Donas et al, (19), 2011 | × | √ | √ | × | × | × | √ |
| Gonzalez et al, (35), 2011 | × | √ | √ | √ | × | × | √ |
| Reichmann et al, (36), 2011 | √ | √ | √ | √ | √ | √ | √ |
| Jost et al, (37), 2012 | √ | √ | √ | √ | √ | × | √ |
| Liistro et al, (38), 2012 | × | √ | √ | √ | × | √ | √ |
| Marcucci et al, (39), 2012 | √ | √ | √ | √ | × | √ | √ |
| Montorsi et al, (40), 2012 | × | √ | √ | √ | × | √ | √ |
| Tekieli et al, (20), 2012 | × | √ | √ | √ | √ | √ | √ |
| Del Giudice et al, (41), 2014 | √ | √ | √ | √ | √ | √ | √ |
| Hynes et al, (42), 2014 | × | × | × | × | √ | × | × |
| Wu et al, (43), 2015 | √ | √ | √ | √ | √ | √ | √ |
| Chung et al, (17), 2016 | √ | √ | √ | √ | √ | √ | √ |
| Columbo et al, (44), 2016 | √ | × | √ | √ | √ | × | √ |
| Moon et al, (45), 2016 | × | √ | √ | √ | √ | √ | √ |
| Nishihori et al, (46), 2016 | × | √ | √ | √ | √ | √ | √ |
| Arhuidese et al, (11), 2017 | √ | √ | × | × | √ | √ | √ |
| Davidovic et al, (47), 2017 | √ | √ | √ | × | × | × | √ |
| Yu et al, (48), 2017 | √ | √ | √ | √ | √ | √ | √ |
| Pohlmann et al, (12), 2018 | √ | √ | √ | √ | √ | √ | √ |
| Tekieli et al, (13), 2019 | × | √ | √ | √ | √ | × | √ |

**Supplementary Table II (online only).** Patient characteristics of the 35 studies included.

| Characteristics | Mean±SD or No. (% of the patients) |
| --- | --- |
| Total patients | 1359 |
| Total interventions for ISR | 1374 |
| Age, years | 68.68±4.79 |
| Gender |  |
| Male | 448 (33%) |
| Female | 470 (35%) |
| Not reported | 441 (32%) |
| Category of ISR |  |
| Symptomatic | 523 (38%) |
| Asymptomatic | 333 (25%) |
| Not reported | 503 (37%) |
| Hypertension | 737/877 (84%) |
| Diabetes mellitus | 340/874 (39%) |
| Hyperlipidemia | 113/224 (50%) |
| Coronary artery disease | 276/780 (35%) |
| Smoker | 291/761 (38%) |
| Contralateral occlusion | 108/736 (15%) |

ISR, in-stent restenosis.

**Supplementary Table III (online only).**Criteria, imaging techniques, and ultrasound criteria used to confirm significant in-stent restenosis (ISR) in 35 studies.

| Study | Degree of ISR | Imaging | Ultrasound criteria for ISR | |
| --- | --- | --- | --- | --- |
|  |  |  | ISR grade | PSV, EDV, and ICA/CCA |
| Chakhtoura et al, (21), 2001 | Asym and Sym ISR ≥ 80% | DUS, confirmed by DSA | NR | |
| Ehringer et al, (22), 2002 | ISR ≥ 70% | DUS, confirmed by DSA | NR | |
| de Borst et al, (23), 2003 | ISR ≥ 50% | DUS | NR | |
| Lal et al, (24), 2003 | ISR ≥ 80% | DUS, confirmed by DSA | ISR ≥ 80% | PSV > 300, EDV > 120, ICA/CCA > 3.2 |
| Levy et al, (25), 2005 | ISR ≥ 80%, Sym ISR | DUS, confirmed by DSA | ISR ≥ 80% | PSV ≥ 330, EDV ≥ 130, ICA/CCA ≥ 3.8 |
| Pokrajac et al, (26), 2005 | ISR > 50% | DUS | NR | |
| Raithel et al, (27), 2005 | ISR > 50% | NR | NR | |
| Setacci et al, (28), 2005 | ISR ≥ 80% | DUS | ISR ≥ 80% | PSV > 300, EDV > 120, ICA/CCA > 3.2 |
| Reimers et al, (29), 2006 | Asym ISR ≥ 70%, Sym ISR ≥ 50% | DUS, confirmed by DSA | NR | |
| Zhou et al, (30), 2006 | ISR ≥ 80% | DUS, confirmed by DSA | ISR ≥ 80% | EDV > 140, ICA/CCA > 4 |
| Younis et al, (31), 2007 | Asym ISR ≥ 80%, Sym ISR ≥ 60% | DUS, confirmed by DSA | NR | |
| Jimenez et al, (32), 2008 | ISR > 70% | DUS/DSA | NR | |
| Juskat et al, (33), 2008 | NR | DUS, confirmed by DSA | NR | |
| Heck, (18), 2009 | ISR ≥ 70% | DUS, confirmed by DSA | ISR > 70% | PSV > 200, EDV > 135, ICA/CCA > 4 |
| Zahn et al, (34), 2010 | Asym ISR ≥ 80%, Sym ISR ≥ 70% | NR | NR | |
| Donas et al, (19), 2011 | ISR > 80% | DUS, confirmed by CTA/MRA | ISR > 80% | PSV ≥ 250, EDV ≥ 120, ICA/CCA ≥ 3.2 |
| Gonzalez et al, (35), 2011 | Sym | DUS, confirmed by DSA/CTA | NR | |
| Reichmann et al, (36), 2011 | ISR > 70% | DUS, CTA/DSA when deemed necessary | ISR ≥ 70% | PSV ≥ 300, EDV ≥ 140, ICA/CCA ≥ 3.8 |
| Jost et al, (37), 2012 | ISR ≥ 80% | DUS/DSA | NR | |
| Liistro et al, (38), 2012 | Asym ISR > 80% | DUS | ISR ≥ 80% | PSV > 300 |
| Marcucci et al, (39), 2012 | ISR > 70% | DUS, confirmed by CTA | ISR > 70% | PSV > 300, EDV > 140, ICA/CCA > 3.8 |
| Montorsi et al, (40), 2012 | ISR > 80% | DUS, confirmed by CTA | ISR > 80% | PSV > 300 |
| Tekieli et al, (20), 2012 | ISR > 70% | DUS, confirmed by DSA | ISR > 70% | PSV ≥ 300, EDV ≥ 90 |
| Del Giudice et al, (41), 2014 | Sym ISR > 80% | DUS, confirmed by CTA | ISR > 80% | PSV ≥ 300 |
| Hynes et al, (42), 2014 | NR | NR | NR | |
| Wu et al, (43), 2015 | Sym ISR > 70% | DUS, confirmed by DSA/CTA | NR | |
| Chung et al, (17), 2016 | ISR ≥ 50% | DUS, DSA, CTA | ISR ≥ 50% | PSV ≥ 225 |
| Columbo et al, (44), 2016 | NR | DUS/CTA | NR | |
| Moon et al, (45), 2016 | Asym ISR > 60%, Sym ISR > 50% | DUS/CTA/MRA, confirmed by DSA | NR | |
| Nishihori et al, (46), 2016 | Asym ISR > 80%, Sym ISR > 50% | DUS, confirmed by DSA | NR | |
| Arhuidese et al, (11), 2017 | More than 70% of patients, ISR > 70% | NR | NR | |
| Davidovic et al, (47), 2017 | Sym ISR > 75% | DUS, confirmed by CTA | NR | |
| Yu et al, (48), 2017 | ISR > 70% | DSA/CTA | NR | |
| Pohlmann et al, (12), 2018 | ISR ≥ 70% | DUS, confirmed by DSA | ISR > 70% | PSV > 300, EDV > 125, ICA/CCA > 4 |
| Tekieli et al, (13), 2019 | ISR ≥ 70% | DUS, confirmed by DSA | ISR ≥ 70% | PSV > 300, EDV > 90 |

Sym, symptomatic; Asym, asymptomatic; ISR, in-stent restenosis; DUS, duplex ultrasonography; DSA, digital subtraction angiography; CTA, CT angiography; MRA, MR angiography; EDV, end diastolic velocity; PSV, peak systolic velocity; ICA/CCA, internal to common carotid artery peak systolic velocity ratio.

**Supplementary Table IV (online only).** Details of outcomes in included studies.

| Study | Article types | Interpretation of outcomes | Other postoperative outcomes, No. | Re-ISR events, No. | Treatment for Re-ISR |
| --- | --- | --- | --- | --- | --- |
| Chakhtoura et al, (21), 2001 | Cohort study | None | NR | 0 | 0 |
| Ehringer et al, (22), 2002 | Cohort study | As is shown in Table 2, two patients who were treated with rCAS both suffered stroke and TIA and multiple Re-ISR events, and one of them died from stroke. | 0 | 3 | 6 rCAS |
| de Borst et al, (23), 2003 | Cohort study | None | 1 transient worsening of pre-existing paresis | 0 | 0 |
| Lal et al, (24), 2003 | Cohort study | During the follow-up, the patient who was treated with rCAS had asymptomatic internal carotid artery occlusion. One of the patients treated with PTA required two additional balloon angioplasty procedures. | NR | 2 | 2 PTA |
| Levy et al, (25), 2005 | Cohort study | The stroke event and one of the Re-ISR events occurred following PTA, while the other Re-ISR event cannot be attributed to a definite treatment because it was not reported in detail. | 0 | 2 | 1 CB-PTA |
| Pokrajac et al, (26), 2005 | Cohort study | The cause and time of death event was not mentioned. | 0 | 2 | 0 |
| Raithel et al, (27), 2005 | Cohort study | This study aimed to report the complications that occurred after CAS, while the outcomes of treatment for ISR were not reported. Only the Re-ISR events were reported; however, they cannot be attributed to a definite treatment because they were not reported in detail. | NR | 8 | 6 CEA, 2 PTFE graft bypass |
| Setacci et al, (28), 2005 | Cohort study | None | 0 | 0 | 0 |
| Reimers et al, (29), 2006 | Cohort study | During the follow-up, one case of asymptomatic Re-ISR was found 3 months after the endovascular procedure, while it cannot be attributed to a definite treatment because it was not reported in detail. | 0 | 1 | 1 PTA |
| Zhou et al, (30), 2006 | Cohort study | Patient No. 2 (treated with CB-PTA) died due to MI at 14 months. Patient No. 1 (treated with PTA) and patient No. 7 (treated with CB-PTA) both developed Re-ISR. | 0 | 2 | 2 CB-PTA |
| Younis et al, (31), 2007 | Cohort study | The Re-ISR event occurred following CB-PTA. | 0 | 1 | 1 SilverHawk atherectomy catheter |
| Jimenez et al, (32), 2008 | Case report | None | 0 | 0 | 0 |
| Juskat et al, (33), 2008 | Cohort study | None | NR | 0 | 0 |
| Heck, (18), 2009 | Cohort study | None | 0 | 1 | 0 |
| Zahn et al, (34), 2010 | Cohort study | As shown in Table II, the total number of ISR cases after CAS was 95, while interventions were not performed in 9 cases and stents were implanted in 53 cases. Therefore, there were 53 cases treated with rCAS and 33 patients treated with PTA. The outcomes were mixed together with patients who did not undergo intervention. Therefore, the outcomes of patients who were treated with interventions cannot be distinguished. No hospital death events occurred. | NR | NR | NR |
| Donas et al, (19), 2011 | Cohort study | The outcomes were not reported in this study. There were 5 patients who suffered from Re-ISR, while they cannot be attributed to a definite treatment because they were not reported in detail. | NR | 5 | 2 vein graft bypass, 6 PTA |
| Gonzalez et al, (35), 2011 | Case report | The postoperative outcomes were only reported in patient No. 3. | NR | 0 | 0 |
| Reichmann et al, (36), 2011 | Cohort study | None | 2 neck hematomas | 0 | 0 |
| Jost et al, (37), 2012 | Case report | All other postoperative outcomes occurred following CEA. | 1 hematoma, 1 Taa with cardiac decompensation, 1 Hypertensive urgency and taa | 0 | 0 |
| Liistro et al, (38), 2012 | Case report | None | NR | 0 | 0 |
| Marcucci et al, (39), 2012 | Cohort study | Patient No. 3, treated with PTFE interposition graft, died due to MI, during the mean follow-up of 18 months (4-36 months). Patient No. 4, treated with PTFE interposition graft, experienced temporary dysfunction of the laryngeal nerve during the perioperative period. | 1 temporary dysfunction of laryngeal nerve | 0 | 0 |
| Montorsi et al, (40), 2012 | Cohort study | This study only reported the long-term overall outcomes of patients treated with DEB-PTA. The details of subjects treated with CB-PTA and rCAS are not available. | NR | 7 | 0 |
| Tekieli et al, (20), 2012 | Cohort study | This study only reported the long-term overall outcomes of 7 patients who suffered Re-ISR events and were treated with DES. | NR | 7 | 7 DES |
| Del Giudice et al, (41), 2014 | Cohort study | One patient died at 3 months from trauma. | 0 | 3 | 3 DEB-PTA |
| Hynes et al, (42), 2014 | Cohort study | The postoperative outcomes are described in the Supplementary materials, while the long-term overall outcomes were not reported in this study. | 0 | NR | NR |
| Wu et al, (43), 2015 | Cohort study | One patient died at 21 months from lung cancer. However, the death and other events cannot be attributed to a definite treatment because they were not reported in detail. | 1 neck hematoma, 1 cerebral hyperperfusion | 0 | 0 |
| Chung et al, (17), 2016 | Cohort study | During the postoperative period, patient No. 1, treated with CB-PTA (described in Supplementary Table II), experienced stroke and subsequent death. In terms of the long-term overall outcomes, two stroke & TIA events and one of the death events occurred following PTA while other evens cannot be attributed to a definite treatment because they were not reported in detail. | 0 | 2 | NR |
| Columbo et al, (44), 2016 | Cohort study | During the postoperative period, there was one patient, treated with bypass, who experienced pneumonia. During the follow-up, there was one patient, treated with bypass, who developed an asymptomatic Re-ISR. He experienced 1 minor ipsilateral stroke at 14.4 months and died of unknown cause at 20 months. The other stroke event and two death events cannot be attributed to a definite treatment because they were not reported in detail. (One patient died of unknown causes after the last follow-up visit at 25 months. One patient died over 10 years after the procedure.) | 1 pneumonia | 1 | 1 PTA |
| Moon et al, (45), 2016 | Cohort study | During the follow-up, 2 symptomatic occlusions and 4 symptomatic restenosis occurred. They cannot be attributed to a definite treatment because they were not reported in detail. | 0 | 6 | 2 ICA-ECA bypass |
| Nishihori et al, (46), 2016 | Cohort study | None | 0 | 0 | 0 |
| Arhuidese et al, (11), 2017 | Cohort study | During the postoperative period, 7 stroke & TIA events, 5 deaths, and 11 other events occurred after rCAS, while 2 stroke & TIA events, 5 deaths, and 9 other events occurred following CEA. As for the long-term overall outcomes, 8 stroke & TIA events occurred after rCAS, and 3 stroke & TIA events occurred following CEA. As for all-cause death events, there were 55 deaths occurred after rCAS and 11 deaths occurred following CEA over the entire study period. | 4 cranial nerve injury, 6 arrhythmias, 9 MI | NR | NR |
| Davidovic et al, (47), 2017 | Case report | None | NR | 0 | 0 |
| Yu et al, (48), 2017 | Cohort study | One patient died 4 years later because of rectal cancer. | 1 hypoglossal nerve dysfunction, 1 dissecting aneurysm, 1 cerebral hyperperfusion | 1 | NR |
| Pohlmann et al, (12), 2018 | Cohort study | Two patients were lost to follow-up due to death: one deceased due to a bronchial carcinoma 11 months after DEB, one deceased due to urosepsis 20 months after the first DEB intervention. | 0 | 1 | 1 DEB |
| Tekieli et al, (13), 2019 | Cohort study | One stroke occurred during the postoperative period, while it cannot be attributed to a definite treatment because it was not reported in detail. There were 13 Re-ISR events; 6 occurred after PTA, 6 occurred following DEB, and 1 occurred after DES. | 0 | 13 | 4 DEB-PTA,,9 DES |

TAA, tachyarrhythmia absoluta/atrial fibrillation.

**Supplementary Table V (online only).** Stent types used for carotid artery stenting (CAS) and repeat CAS.

| Study | CAS | Repeat CAS |
| --- | --- | --- |
| Chakhtoura et al, (21), 2001 | self-expanding Wallstents ^a^, Palmaz P-154 ^b^ | Palmaz stent |
| Ehringer et al, (22), 2002 | Wallstent ^c^ | NR |
| de Borst et al, (23), 2003 | carotid wall, easy wall | NR |
| Lal et al, (24), 2003 | WallStents ^a^,Acculink ^d^ | Palmaz stent ^e^ |
| Setacci et al, (28), 2005 | WallStent ^a^ | NR |
| Reimers et al, (29), 2006 | NR | Wallstent ^c^ |
| Zhou et al, (30), 2006 | Wallstent ^c^, Acculink ^f^ | NR |
| Younis et al, (31), 2007 | PRECISE ^g^, Wallstent ^c^, SMART ^g^ | Acculink ^h^ |
| Jimenez et al, (32), 2008 | Wallstent ^c^ | NR |
| Juskat et al, (33), 2008 | NR | self-expanding Smart Precise stent ^i^ |
| Donas et al, (19), 2011 | Wallstent, Acculink, Exponent | Wallstent |
| Reichmann et al, (36), 2011 | Cristallo Ideale ^j^, Carotid Wall Stent ^c^, Palmaz stents ^e^, Precise stents ^e^, Acculink ^k^ | NR |
| Liistro et al, (38), 2012 | Wallstent | NR |
| Marcucci et al, (39), 2012 | Wallstent ^c^, Xact ^k^ | NR |
| Montorsi et al, (40), 2012 | NR | Wallstent ^c^ |
| Tekieli et al, (20), 2012 | Wallstent, Cristallo Ideale, Xact, Acculink | Zotarolimus-eluting stent |
| Del Giudice et al, (41), 2014 | Wallstents ^c^, Rx Acculink stents ^l^ | NR |
| Chung et al, (17), 2016 | Wallstenta ^m^, Xact ^n^, Protégé ^o^ | Wallstenta ^m^, Xact ^n^ |
| Columbo et al, (44), 2016 | Wallstent ^c^, Acculink ^n^, Precise ^i^ | NR |
| Nishihori et al, (46), 2016 | Wallstent ^c^, PRECISE | PRECISE, Wallstent ^c^ |
| Arhuidese et al, (11), 2017 | NR | NR |
| Davidovic et al, (47), 2017 | NR | Wallstents ^c^ |

^a^Meditech/Boston Scientific, Minneapolis, Minn; ^b^Johnson and Johnson Interventional Systems Co, Warren, NJ; ^c^Boston Scientific, Natick, MA, USA; ^d^Guidant, Menlo Park, Calif; ^e^Cordis, Miami Lakes, Fla; ^f^Guidant, Santa Clara, Calif; ^g^Cordis, Miami, FL; ^h^Guidant, Indianapolis, MD; ^i^Cordis; ^j^Invatec SPA, Roncadelle, Italy; ^k^Abbott, Abbott Park, Ill; ^l^Abbott Laboratories, Abbott Park, IL, USA; ^m^Boston Scientific, Marlborough, Mass; ^n^Abbott Vascular, Santa Clara, Calif; ^o^ev3 Endovascular Inc, Plymouth, Minn

**Supplementary Table VI (online only).** Summary of studies with intact basic characteristics.

| Study | Pts, No. | Sym ISR, No. | Time to ISR | ISR treatment, No. | Postoperative outcomes | | Follow-up time | long-term overall outcomes | | Re-ISR, No. |
| --- | --- | --- | --- | --- | --- | --- | --- | --- | --- | --- |
|  |  |  |  |  | Stroke & TIA | Death |  | Stroke & TIA | Death |  |
| Chakhtoura et al, (21), 2001 | 4 | 0 | 13 m | 3 PTA, 1 rCAS | NR | 0 | 10 m | 0 | 0 | 0 |
| Ehringer et al, (22), 2002 | 8 | 1 | 6.7 m | 6 rCAS, 3 PTA | 0 | 0 | NR | 2 | 1 | 3 |
| Setacci et al, (28), 2005 | 14 | 5 | 39 m | 3 PTA, 4 CB-PTA, 8 rCAS | 0 | 0 | 12.4 m | 0 | 0 | 0 |
| Reimers et al, (29), 2006 | 31 | 7 | 5.3 m | 12 PTA, 10 CB-PTA, 10 rCAS | 0 | 0 | 17m | 0 | 0 | 1 |
| Zhou et al, (30), 2006 | 7 | 1 | 14 m | 4 CB-PTA, 1 PTA, 2 rCAS | 0 | 0 | 9 m | NR | 1 | 2 |
| Reichmann et al, (36), 2011 | 15 | 10 | 18.3 m | 15 CEA | 1 | 0 | 21 m | 1 | 0 | 0 |
| Jost et al, (37), 2012 | 3 | NR | 20.5 w | 3 CEA, 1 Dacron graft bypass | 0 | 0 | 11.5 m | 0 | 0 | 0 |
| Marcucci et al, (39), 2012 | 7 | 3 | 13.1 m | 5 CEA, 2 PTFE graft bypass | 0 | 0 | 18 m | 0 | 1 | 0 |
| Del Giudice et al, (41), 2014 | 9 | 9 | 3.6 m | 9 DEB-PTA | 0 | 0 | 36.6 m | 0 | 1 | 3 |
| Wu et al, (43), 2015 | 21 | 21 | 15.7 m | 15 CEA, 6 PTFE graft bypass | 0 | 0 | 13.2 m | 0 | 1 | 0 |
| Chung et al, (17), 2016 | 30 | NR | NR | 19 PTA, 21 rCAS | 1 | 1 | 948 d | 2 | 9 | 2 |
| Columbo et al, (44), 2016 | 8 | 4 | NR | 5 CEA, 3 bypass | 0 | 0 | 38.7 m | 2 | 3 | 1 |
| Arhuidese et al, (11), 2017 | 645 | 426 | NR | 511 rCAS, 134 CEA | 9 | 10 | 1 y | 11 | 66 | NR |
| Davidovic et al, (47), 2017 | 4 | 4 | 26 m | 4 bypass: 2 PTFE graft, 2 Dacron graft | NR | NR | 13 m | 0 | 0 | 0 |
| Yu et al, (48), 2017 | 10 | 9 | 6.5 m | 10 CEA | 0 | 0 | 25 m | 0 | 1 | 1 |
| Pohlmann et al, (12), 2018 | 9 | 1 | 9 m | 10 DEB-PTA | 0 | 0 | 5 y | 1 | 2 | 1 |

**Supplementary Table VII (online only).** Comparison of outcomes in the PTA, rCAS, and CEA groups in studies with intact basic characteristics.

| Outcome | PTA | rCAS | CEA | PTA vs. rCAS | | PTA vs. CEA | | rCAS vs. CEA | |
| --- | --- | --- | --- | --- | --- | --- | --- | --- | --- |
|  |  |  |  | χ2 | *P* | χ2 | *P* | χ2 | *P* |
| **Postoperative outcomes** | | | | | | | | | |
| Stroke & TIA | 1/75 (1.3%) | 7/558 (1.3%) | 3/188 (1.6%) | - | 1 | - | 1 | 0 | 1 |
| Death | 1/78 (1.3%) | 5/559 (0.9%) | 3/188 (1.6%) | 0 | 1 | - | 1 | 0.160 | 0.690 |
| **Long-term overall outcomes** | | | | | | | | | |
| Stroke & TIA | 3/71 (4.2%) | 10/557 (1.8%) | 4/182 (2.2%) | 0.831 | 0.362 | 0.209 | 0.648 | 0.001 | 0.974 |
| Death | 5/59 (8.5%) | 56/539 (10.4%) | 12/167 (7.2%) | 0.213 | 0.644 | 0.001 | 0.972 | 1.504 | 0.220 |
| Re-ISR | 7/53 (13.2%) | 3/38 (7.9%) | 1/53 (1.9%) | 0.211 | 0.646 | - | 0.060 | - | 0.304 |

**Supplementary Table VIII (online only).** Comparison of baseline characteristics of patients in the PTA, rCAS, and CEA groups.

| Characteristics | PTA | rCAS | CEA | PTA vs. rCAS | | PTA vs. CEA | | rCAS vs. CEA | |
| --- | --- | --- | --- | --- | --- | --- | --- | --- | --- |
|  |  |  |  | χ^2^ | *P* | χ^2^ | *P* | χ^2^ | *P* |
| Symptomatic ISR | 19/59 (32%) | 345/530 (65%) | 114/174 (66%) | 24.33 | **<0.001** | 19.96 | **<0.001** | 0.01 | 0.919 |
| Hypertension | 14/18 (78%) | 467/511 (91%) | 146/167 (87%) | 2.43 | 0.119 | 0.60 | 0.439 | 2.28 | 0.131 |
| Diabetes mellitus | 10/18 (56%) | 196/511 (38%) | 84/166 (51%) | 2.16 | 0.141 | 0.16 | 0.690 | 7.75 | **0.005** |
| Hyperlipidemia | 15/18 (83%) | NR | 20/28 (71%) | - | - | 0.33 | 0.569 | - | - |
| Coronary artery disease | 6/15 (40%) | 168/512 (33%) | 64/967 (7%) | 0.09 | 0.760 | 20.08 | **<0.001** | 173.67 | **<0.001** |
| Smoker | 11/18 (61%) | 156/511 (31%) | 63/158 (40%) | 7.53 | **0.006** | 2.99 | 0.084 | 4.79 | **0.029** |
| Contralateral occlusion | 6/12 (50%) | 73/512 (14%) | 18/168 (11%) | 9.07 | **0.003** | 11.75 | **0.001** | 1.37 | 0.242 |
